# Supplementary material for: Genome editing reveals fitness effects of a gene for sexual dichromatism in Sulawesian fishes
Source: Nat Commun. 2021 Mar 1;12:1350. doi: 10.1038/s41467-021-21697-0 (PMC7921647; doi:10.1038/s41467-021-21697-0)
Supplement: Supplementary file 2 — Supplementary Information [file 41467_2021_21697_MOESM2_ESM.pdf]

**Supplementary Information:**

Genome editing reveals fitness effects of *csf1* gene responsible for sexual dichromatism in Sulawesian fishes

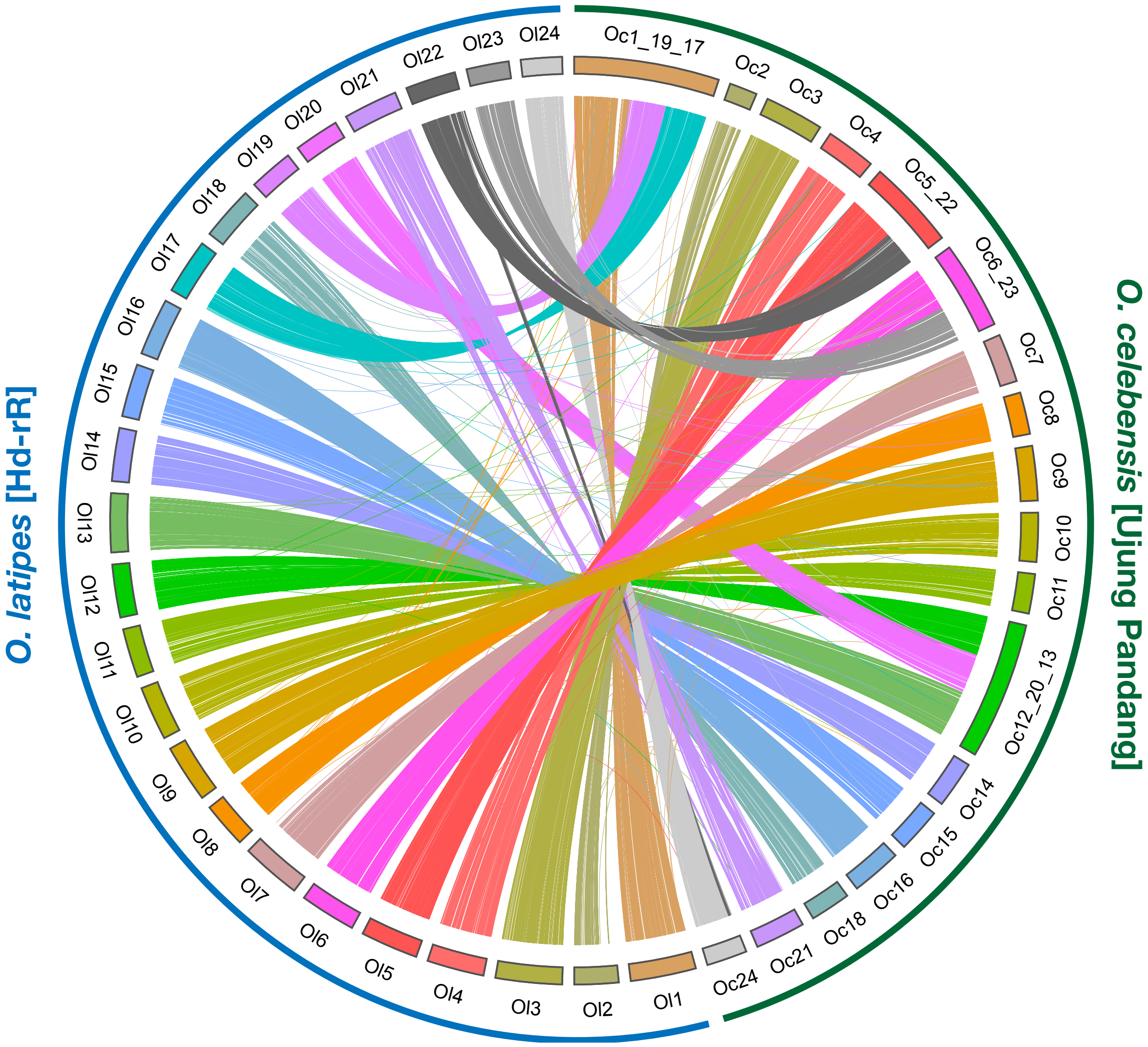


**Supplementary Figure 1 | Chromosomal rearrangements in *Oryzias celebensis*.**
A circos plot showing homologous gene pairs across the reference assemblies of *O. celebensis* [Ujung pandang] and *O. latipes* [Hd-rR]. Reciprocal BLAST analysis by OrthoFinder 2.2.6 identified 13,777 single orthologs. Lines connect each orthologous gene pair. The different colors indicate genes on each chromosome of *O. latipes*.

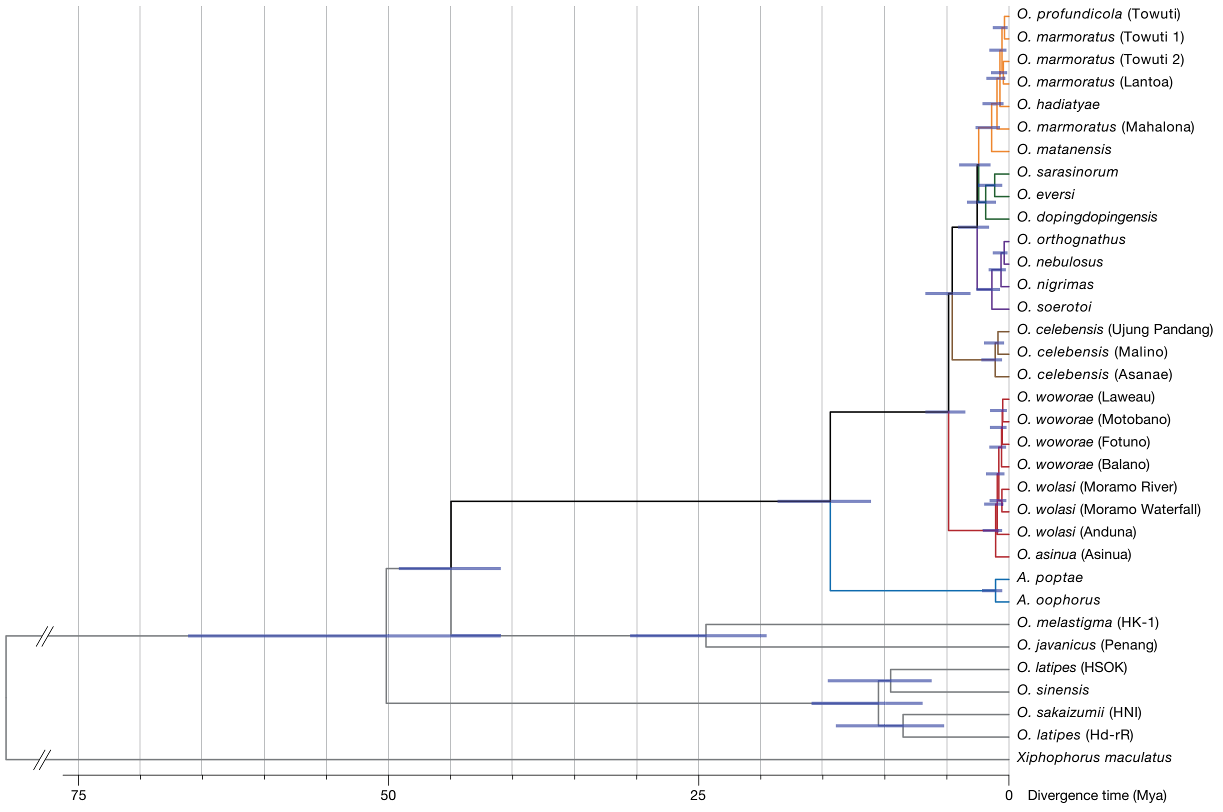


**Supplementary Figure 2 | Chronogram showing estimated divergence times for Adrianichthyidae species.**
Lineage divergence times were estimated with RelTime using 500 clock-like genes. The X-axis indicates the time scale in millions of years ago (Mya). The blue bars at nodes indicate 95% confidence intervals for mean estimated time. The major lineages of Sulawesi species are shown as different colors.

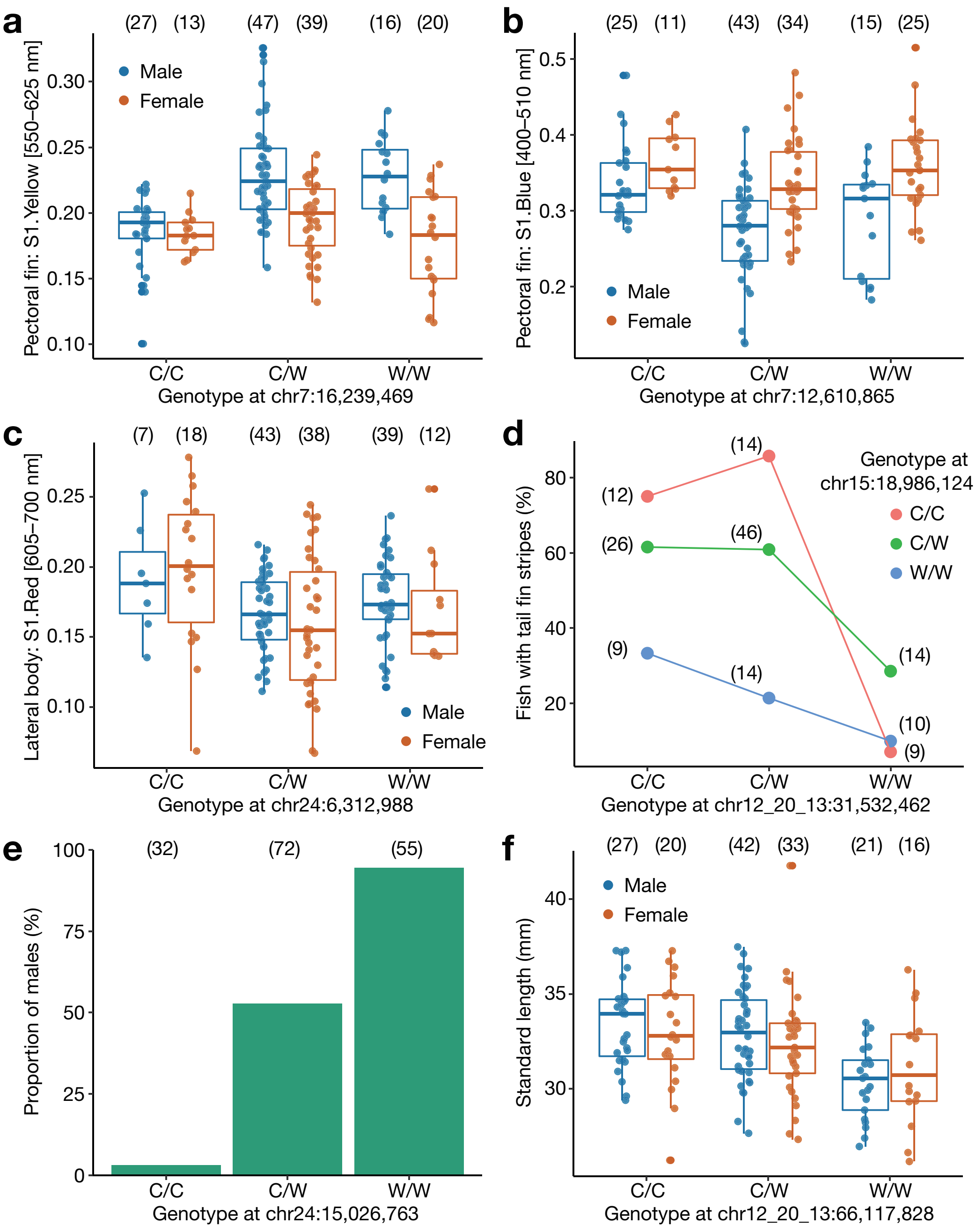


**Supplementary Figure 3 | Effect plots for significant QTLs.**
**a**, **b**, The relative contribution of the yellow (550–625 nm) (**a**) or blue (400–510 nm) (**b**) spectral ranges to the total brightness in the pectoral fins of the $F_2$ family. **c**, The relative contribution of the red spectral range (605–700 nm) to the total brightness in the lateral body surface. **d**, Percentages of fish with black stripes in the tail fin. **e**, Proportion of males out of the total fish with each genotype near the QTL of sex. **f**, Standard length (mm) of the $F_2$ fish. In all panels, the X-axis indicates genotypes at each QTL. In **d**, different colors indicate genotypes at the second significant QTL. C/C, homozygous for the *O. celebensis* allele; W/W, homozygous for the *O. woworae* allele; C/W, heterozygous for both alleles. In **a**–**c** and **f**, each dot represents each individual, the center line indicates the median, box limits indicate the upper and lower quartiles, the whiskers indicate 1.5x interquartile range, and the points are outliers. The blue and brown colors indicate males and females, respectively. Sample sizes are shown in the parentheses. Source data are provided as a Source Data file.

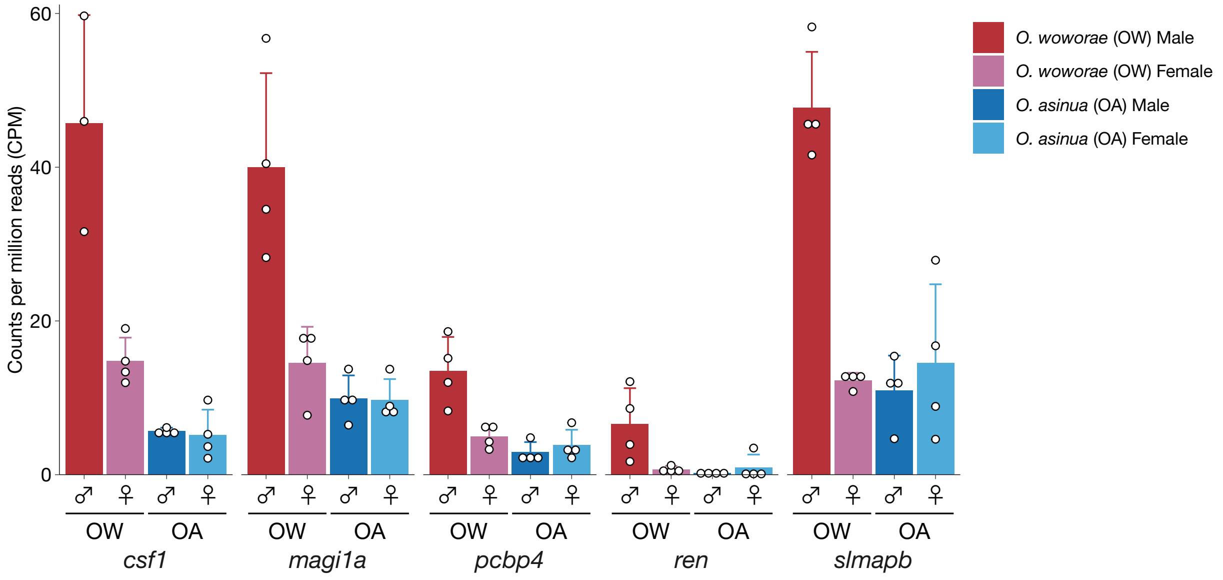


**Supplementary Figure 4 | Expression levels of candidate genes for the red pectoral fins.** Counts of transcripts per million reads (CPM) were extracted from the RNA-seq data of the pectoral fins ($n$ = 4 in each group). All five genes (*csf1*, *magi1a*, *pcbp4*, *ren*, and *slmapb*) are located within the red pectoral fin QTLs and expressed at higher levels in *O. woworae* males, but none of these genes except *csf1* are known to be expressed or function in pigment cells. To compare the expression level of *csf1* gene with those of the other genes, CPM of *csf1* (see Figure 3D) is also shown here. Mean ± SD are shown. Each circle represents each individual. The red, pink, blue, and light blue colors indicate *O. woworae* (OW) males, OW females, *O. asinuia* (OA) males, and OA females, respectively. Source data are provided as a Source Data file.

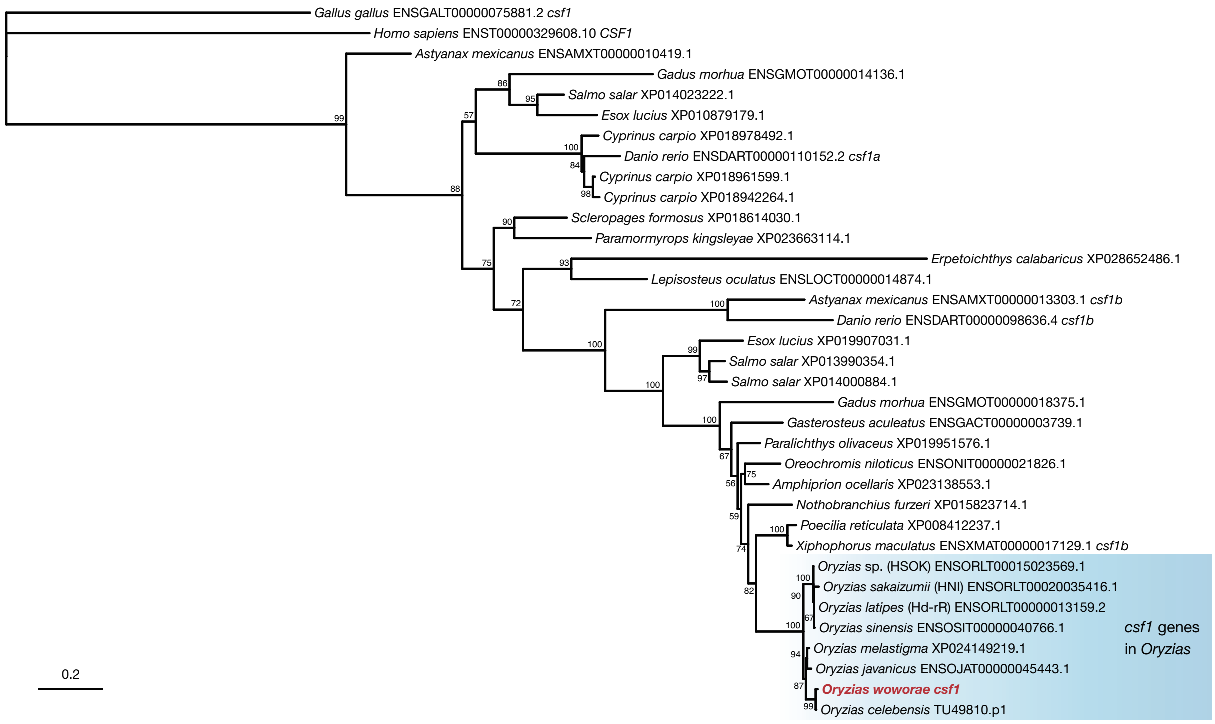


**Supplementary Figure 5 | A phylogenetic tree of the *csf1* gene in Actinopterygii.**
A maximum-likelihood phylogenetic tree inferred using a 684-bp of alignment of *csf1*-coding sequences from 24 Actinopterygian species and two non-Actinopterygian vertebrates (*Homo sapiens* and *Gallus gallus*). The scale bar indicates the substitution rate. The number beside each node indicates the maximum likelihood bootstrap values. The genes of *Oryzias* species and the focal gene of *Oryzias woworae* are highlighted in a blue box and red letters, respectively.

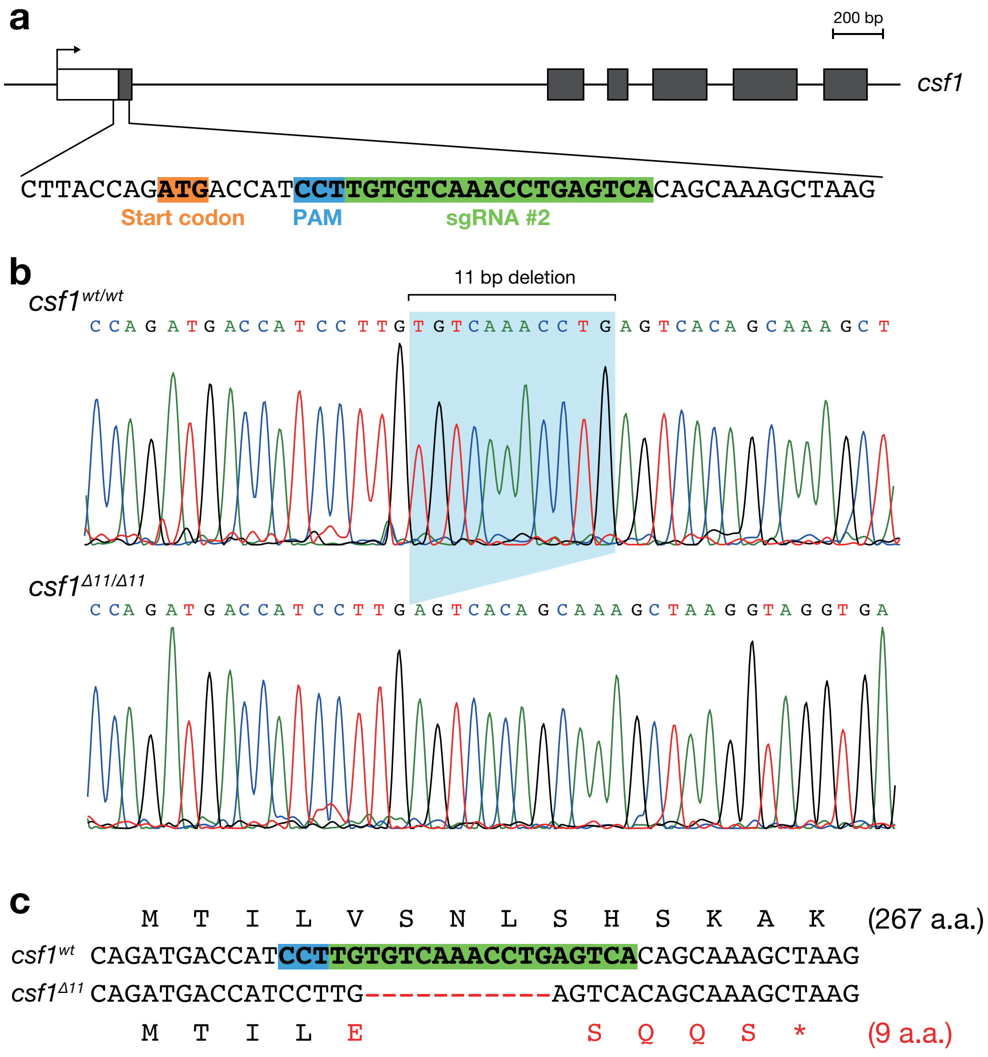


**Supplementary Figure 6 | Targeted knock-out of the *csf1* gene using the CRISPR/Cas system.** **a**, A schematic illustration of the design of a sgRNA (sgRNA#2) targeting the *O. woworae csf1* gene. Gray and white boxes indicate translated and untranslated exon sequences, respectively. The nucleotide sequence with an orange box indicates the putative start codon of *csf1* gene. **b**, Representative chromatograms of Sanger sequencing of the *csf1* wild-type fish (*csf1*$^{wt/wt}$) and the homozygous knock-out fish (*csf1*$^{\Delta 11/\Delta 11}$). In the knock-out fish, a 11-bp region (highlighted by a blue shade on the wild-type image) was deleted. **c**, The deletion (*csf1*$^{\Delta 11}$) will produce a truncated protein (four correct amino acids followed by five amino acids with missense mutations) while the wild-type allele (*csf1*$^{wt}$) can produce an intact protein of 267 amino acids. In **a** and **c**, the nucleotide sequences highlighted with blue and green boxes show the protospacer adjacent motif (PAM) and the recognition sequence for the sgRNA target, respectively.

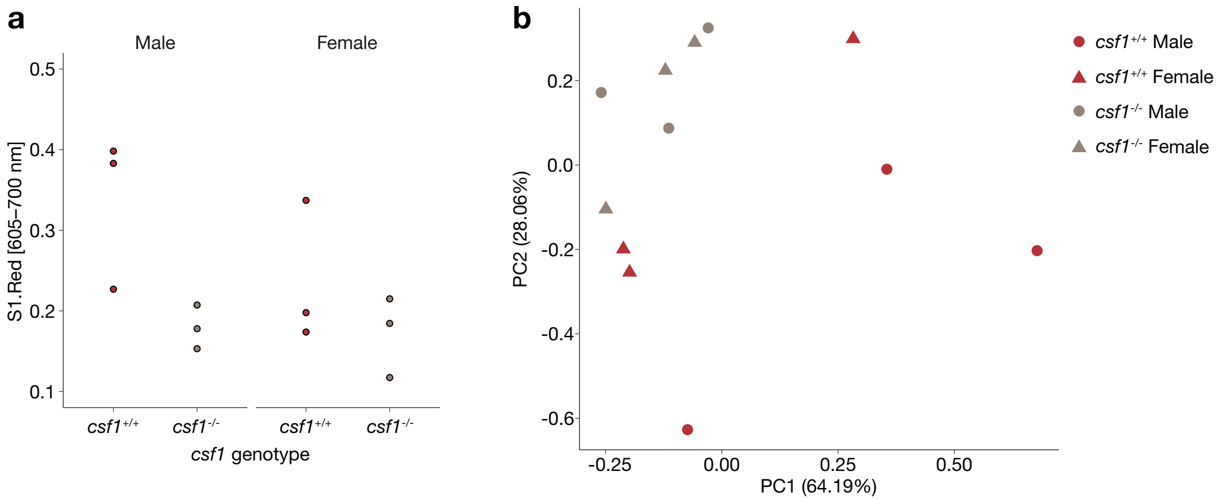


**Supplementary Figure 7 | Effects of the *csf1* knock-out on the reflectance spectra in the pectoral fin.**

The reflectance of each pectoral fin was measured in both sexes of *csf1* wild-type (*csf1*$^{+/+}$) or knock-out (*csf1*$^{-/-}$) ($n$ = 3 for each). **a**, The relative contributions of a red spectral range (605–700 nm wavelengths) to the total brightness (S1.Red). The *csf1* knock-out significantly decreases the contribution (two-way ANOVA, *csf1* genotype: estimate ± s. e. = -0.11029 ± 0.04145, $t$ = -2.660, $P$ = 0.026; sex: estimate ± s. e. = -0.05350 ± 0.04145, $t$ = -1.290, $P$ = 0.229). **b**, Principal component analysis (PCA) indicates that the *csf1* mutation reduces sexual difference in the spectral patterns. Each dot represents a single individual. The closed circles and triangles indicate males and females, respectively. The red and brown colors indicate *csf1* wild-type and knock-out fish, respectively. Source data are provided as a Source Data file.

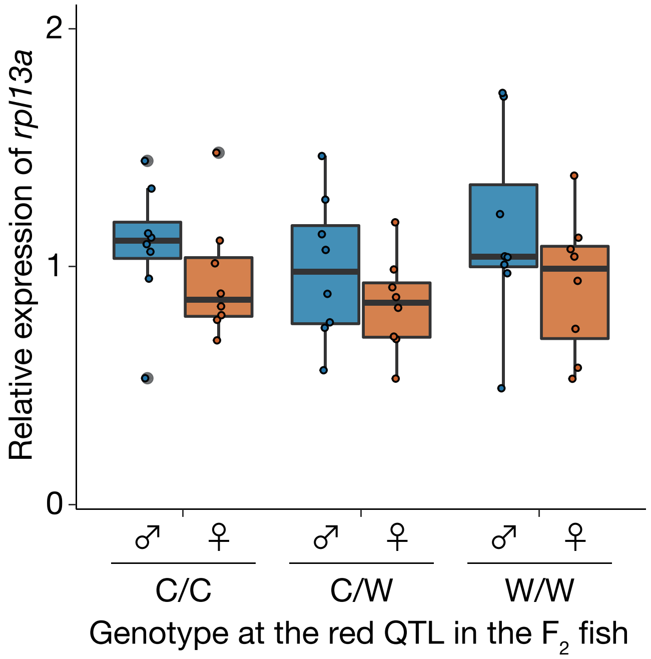


**Supplementary Figure 8 | Expression levels of the *rpl13a* gene as an internal control in the $F_2$ family for QTL analysis.**
Relative expression levels standardized with serially diluted cDNA pools of all analyzed samples are shown ($n$ = 8 in each group). The X-axis indicates genotypes at the QTL for the red pectoral fin on LG7 shown separately for each sex (C/C, homozygous for the *O. celebensis* allele; W/W, homozygous for the *O. woworae* allele; C/W, heterozygous for both alleles). No significant difference among genotypes was found using two-way ANOVA (genotype: $F_{2,44}$ = 0.840, $P$ = 0.4534, sex: $F_{1,44}$ = 4.181, $P$ = 0.0516). Each dot represents each individual, the center line indicates the median, box limits indicate the upper and lower quartiles, the whiskers indicate 1.5x interquartile range, and the points are outliers. The blue and red colors indicate males and females, respectively. Source data are provided as a Source Data file.

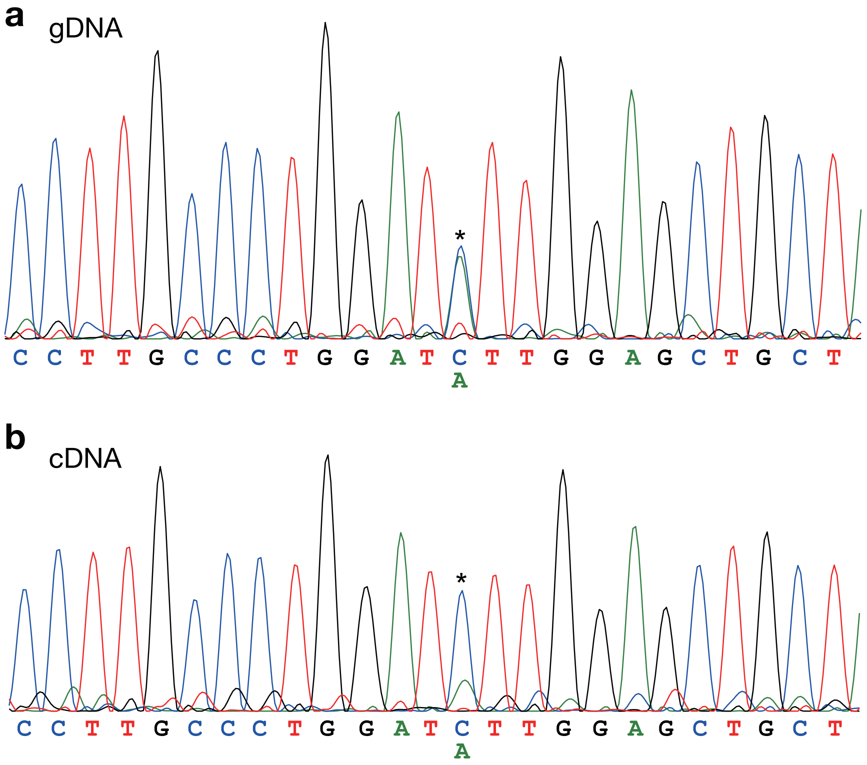


**Supplementary Figure 9 | Chromatograms for allele-specific expression analysis of *csf1*.** Representative chromatograms of Sanger sequencing of the *csf1* gene from a single $F_2$ hybrid individual (heterozygote of *O. woworae* and *O. celebensis* alleles at *csf1*) using the genomic DNA (gDNA) (**a**) or the cDNA of the pectoral fins (**b**) as a template. At a variant site (indicated by asterisks), signal ratios between the “A” allele (from *O. celebensis*) and the “C” allele (from *O. woworae*) were quantified as described in Methods. Each nucleotide base is shown as different colors.

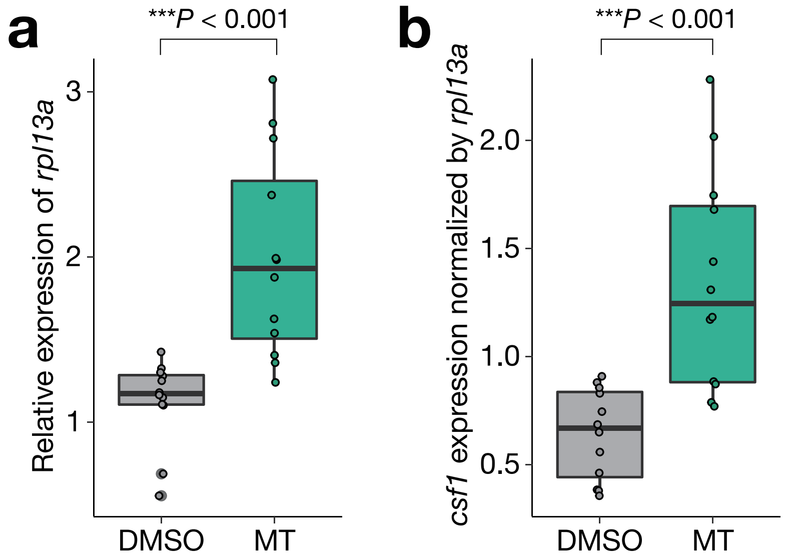


**Supplementary Figure 10 | Expression levels of the *rpl13a* gene in the pectoral fins of *O. woworae* females with methyltestosterone (MT) administration.**
**a**, Relative expression levels of the *rpl13a* gene standardized with serially diluted cDNA pools of all analyzed samples are shown ($n$ = 12 for each). The MT administration significantly increased *rpl13a* expression (two-sided Welch's $t$-test, $t_{14.704}$ = -4.5308, $P$ = 0.00042). **b**, Even after normalization with *rpl13a* expression levels, the relative expression levels of the *csf1* gene were higher in the MT-administered fish than in the control fish with dimethyl sulfoxide (DMSO) administration (two-sided Welch's $t$-test, $t_{14.721}$ = -4.5046, $P$ = 0.00044). Each dot represents each individual, the center line indicates the median, box limits indicate the upper and lower quartiles, the whiskers indicate 1.5x interquartile range, and the points are outliers. The grey and green colors indicate fish with DMSO and MT, respectively. Source data are provided as a Source Data file.

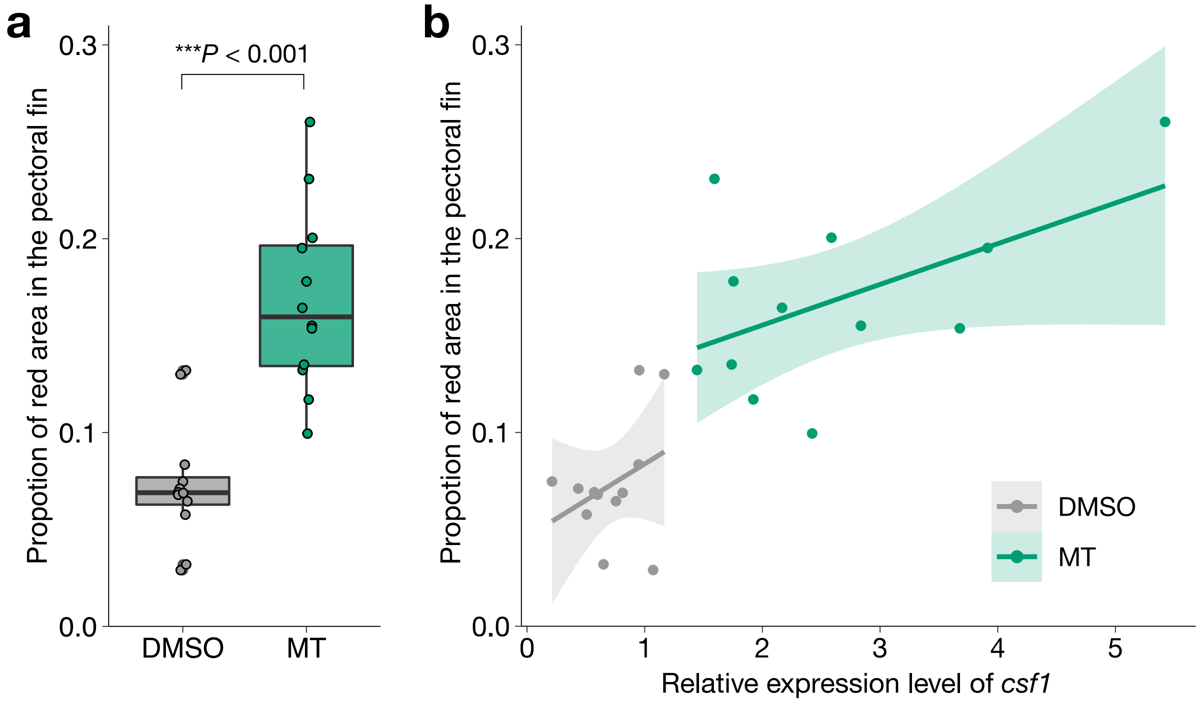


**Supplementary Figure 11 | Effects of methyltestosterone (MT) administration on redness of the pectoral fins in *O. woworae* females.**
**a**, Proportion of red pigmented area in the pectoral fins of *O. woworae* females administrated with dimethyl sulfoxide (DMSO) or MT ($n$ = 12 for each). The MT administration significantly increased the red area in the pectoral fins (two-sided Welch's $t$-test, $t_{19.166}$ = -5.8209, $P$ = 1.272 x $10^{-5}$). Each dot represents each individual, the center line indicates the median, box limits indicate the upper and lower quartiles, the whiskers indicate 1.5x interquartile range, and the points are outliers. **b**, Both redness and *csf1* expression levels were increased by MT administration. A linear model shows that fish with higher expression levels of *csf1* have larger red area in the pectoral fins (*csf1* expression levels: estimate ± s. e. = 0.02189 ± 0.008967, $t$ = 2.441, $P$ = 0.023582; MT administration: estimate ± s. e. = 0.05350 ± 0.02254, $t$ = 2.374, $P$ = 0.027228). Each dot represents a single individual. Each line shows linear regression with 95% confidence intervals in each treatment. The grey and green colors indicate fish with DMSO and MT, respectively. Source data are provided as a Source Data file.

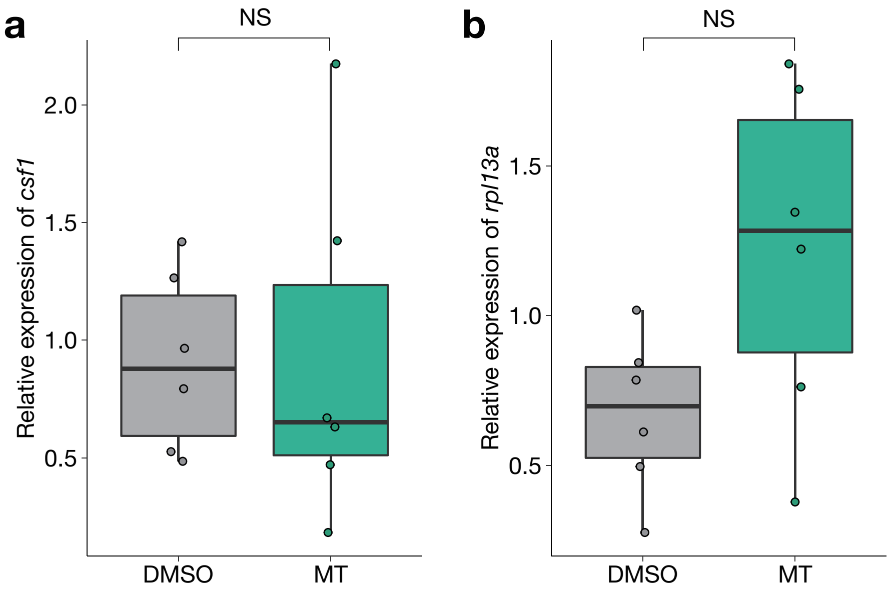


**Supplementary Figure 12 | Effects of methyltestosterone (MT) administration on *csf1* and *rpl13a* expression levels in the pectoral fins of females of *O. asinua*.**
*O. asinua* is a closely related species of *O. woworae* and lacks red coloration in the pectoral fins in both sexes. **a**, **b**, Relative expression levels of *csf1* (**a**) and *rpl13a* (**b**) standardized with serially diluted cDNA pools of all analyzed samples are shown ($n$ = 6 for each). **a**, Relative expression levels of *csf1*. The MT administration did not affect *csf1* (Welch's $t$-test, $t_{7.5007}$ = -0.048346, $P$ = 0.9627) or *rpl13a* (Welch's $t$-test, $t_{7.0979}$ = -2.1347, $P$ = 0.06964) expression in *O. asinua* females. Each dot represents a single individual, the center line indicates the median, box limits indicate the upper and lower quartiles, the whiskers indicate 1.5x interquartile range, and the points are outliers. The grey and green colors indicate fish with DMSO and MT, respectively. NS, not significant according to Welch's $t$-test. Source data are provided as a Source Data file.

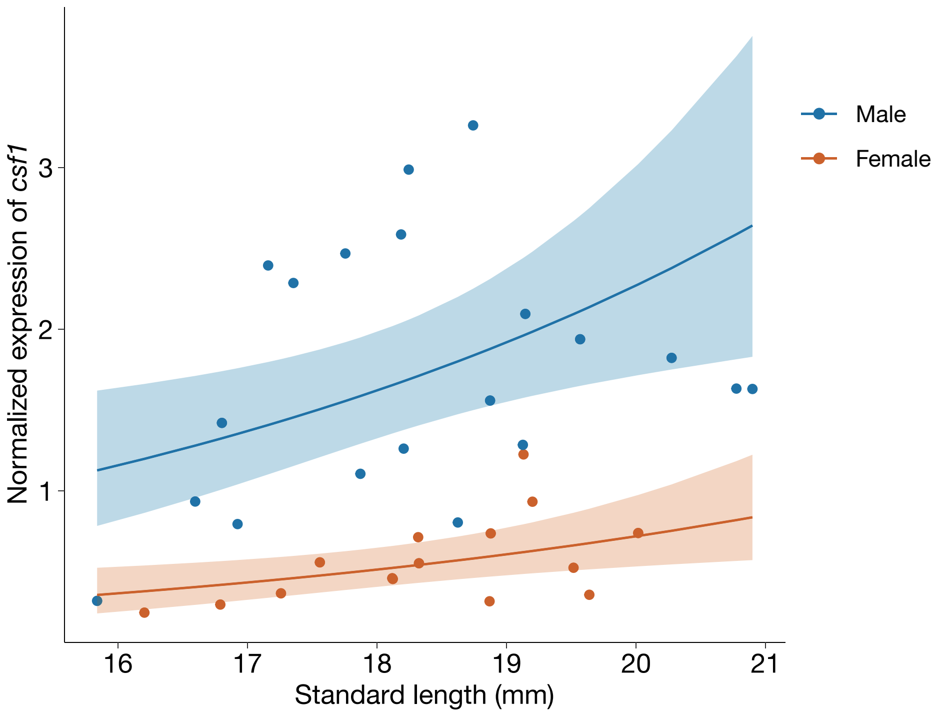


**Supplementary Figure 13 | Relationship between standard length and expression levels of *csf1* in pectoral fins of *O. woworae*.**
Expression levels of *csf1* were quantified in 3-4 month-old fish ($n$ = 20 for male and $n$ = 15 for female). Relative expression levels of *csf1* were normalized by the relative expression levels of an internal control gene *rpl13a*. A generalized liner model (GLM) using a Gamma distribution with a log link function showed that larger fish had significantly higher expression levels of *csf1* in males (standard length: estimate ± s. e. = 0.16835 ± 0.06198, $t$ = 2.716, $P$ = 0.0106; sex: estimate ± s. e. = -1.14881 ± 0.15377, $t$ = -7.471, $P = 1.67 \times 10^{-8}$). Each dot represents a single individual. Each line shows gamma regression with 95% confidence intervals in each sex. The blue and brown colors indicate males and females, respectively. Source data are provided as a Source Data file.

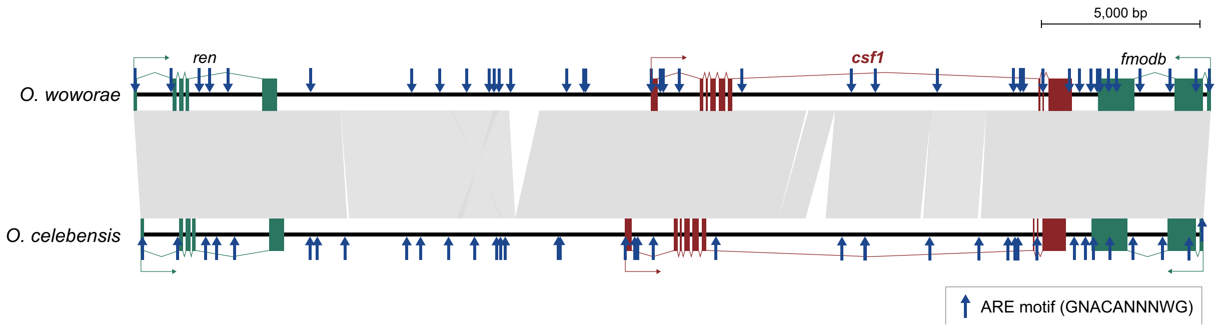


**Supplementary Figure 14 | Androgen response elements (AREs) at the *csf1* locus in *O. woworae* and *O. celebensis*.**

34 kb genomic sequences including *csf1* and two neighboring genes (*ren* and *fmodb*) were extracted from the genome assemblies of *O. woworae* and *O. celebensis*. Arrows indicate the identified positions of AREs (GNACANNNWG; MA0007.2 in JASPAR). There are 11 and 12 AREs in the upstream intergenic regions of *csf1*, 3 and 2 in the exon, 10 and 12 in the intron, and 3 and 2 in the downstream intergenic regions in *O. woworae* and *O. celebensis*, respectively. Colored boxes indicate exon sequences of predicted genes (Red, *csf1*; Green, other genes). Gray areas connecting the two assemblies show syntenic blocks identified with BLAST.

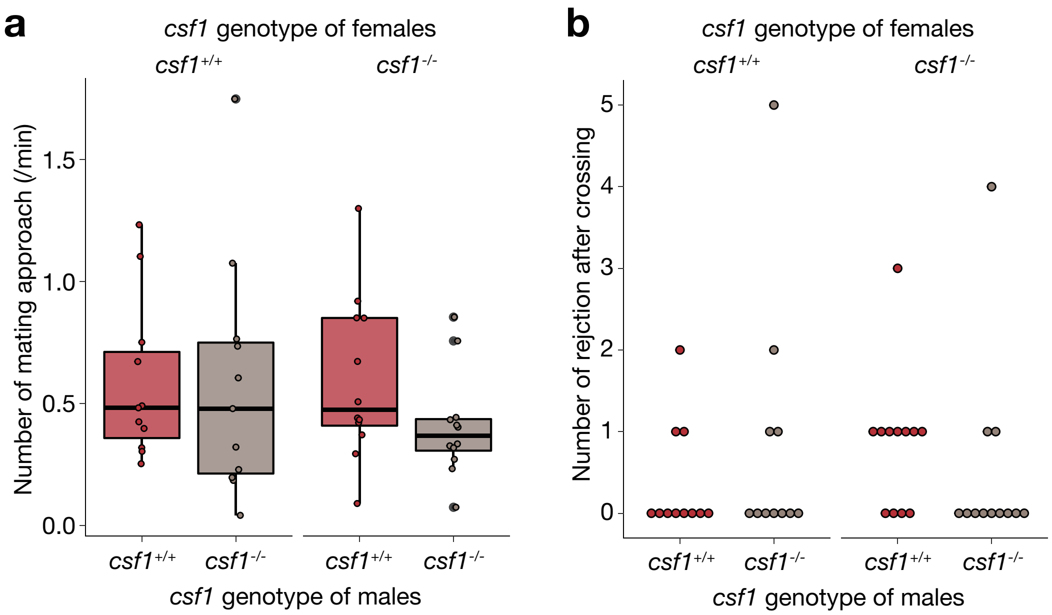


**Supplementary Figure 15 | Mating behaviors of *csf1* wild-type (*csf1*$^{+/+}$) and knock-out (*csf1*$^{-/-}$) *O. woworae*.**
The mating behaviors were observed in 46 pairs in total: 11 pairs between *csf1*$^{+/+}$ female and *csf1*$^{+/+}$ male, 11 pairs between *csf1*$^{+/+}$ female and *csf1*$^{-/-}$ male, 12 pairs between *csf1*$^{-/-}$ female and *csf1*$^{+/+}$ male, 12 pairs between *csf1*$^{-/-}$ female and *csf1*$^{-/-}$ male). **a**, Number of mating approaches per minute across the entire behavioral assay. No significant difference between *csf1* genotypes was found in either sex with the Wald test in a GLMM (male genotype: estimate ± s. e. = -0.16521 ± 0.16404, $z$ = -1.007, $P$ = 0.314; female genotype: estimate ± s. e. = 0.02276 ± 0.19927, $z$ = 0.114, $P$ = 0.909; interaction: estimate ± s. e. = -0.22625 ± 0.25466, $z$ = -0.888, $P$ = 0.374). Each single dot represents a behavioral experiment, the center line indicates the median, box limits indicate the upper and lower quartiles, the whiskers indicate 1.5x interquartile range, and the points are outliers. **b**, Total number of rejections by females after being held by the male. The Wald test in a GLMM showed no significant difference among *csf1* genotypes in either sex (male genotype: estimate ± s. e. = 0.8109 ± 0.6009, $z$ = 1.350, $P$ = 0.1772; female genotype: estimate ± s. e. = 0.8535 ± 0.6316, $z$ = 1.351, $P$ = 0.1766; interaction: estimate ± s. e. = -1.3217 ± 0.7923, $z$ = -1.668, $P$ = 0.0953). A single dot represents one behavioral experiment. The red and grey colors indicate *csf1* wild-type and knock-out fish, respectively. Source data are provided as a Source Data file.

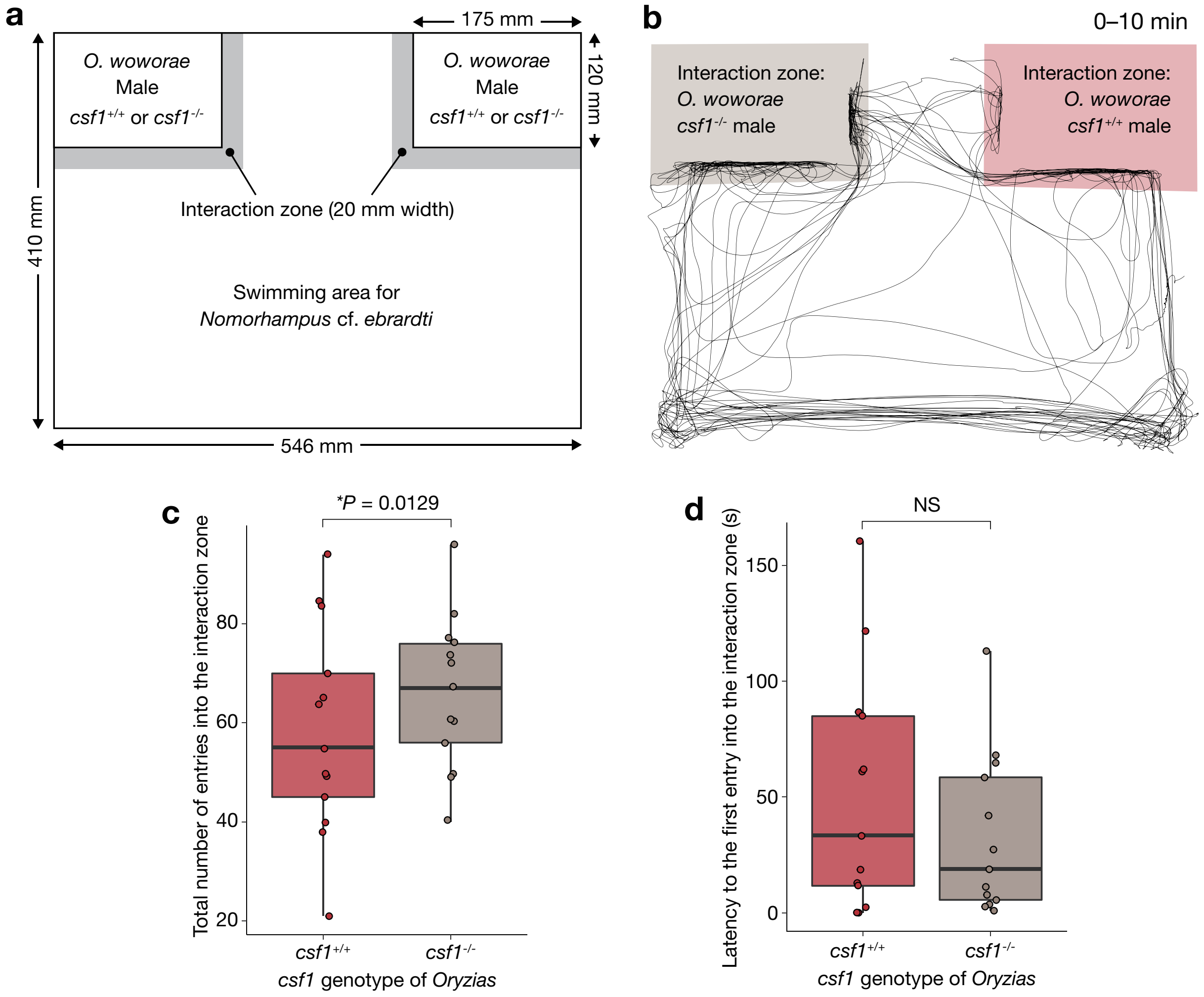


**Supplementary Figure 16 | A behavioral test to assess the effects of the *csf1* genotype of *O. woworae* on predator attraction.**

**a**, Schematic illustration of an apparatus used for the behavioral analysis. An *O. woworae* male with the *csf1* wild-type genotype (*csf1*$^{+/+}$) and a male with the knock-out genotype (*csf1*$^{-/-}$) were put into two separate plastic tanks that were attached to the inside of the testing tank. A predator (halfbeak, *Nomorhamphus* cf. *ebrardti*) was released into the center of the swimming area. The interaction zone was defined as an area within 20 mm from walls of the plastic tank with medaka. **b**, A representative trajectory of a halfbeak during a 10-min behavioral test. The interaction zones are shown in gray in the figure. **c**, Number of entries into each interaction zone by halfbeaks during the first 10-min half of a 20-min behavioral test ($n$ = 13 in halfbeaks). The halfbeaks entered the zone of *csf1*$^{-/-}$ medaka more often than that of *csf1*$^{+/+}$ medaka (GLMM, medaka *csf1* genotype: estimate ± s. e. = 0.12361 ± 0.04971, $t$ = 2.487, $P$ = 0.0129). **d**, Latency to entry into each interaction zone ($n$ = 13 in halfbeaks). No significant difference was found between the interaction zones (GLMM, medaka *csf1* genotype: estimate ± s. e. = -0.4343 ± 0.7341, $t$ = -0.592, $P$ = 0.554). In **c** and **d**, the red and grey colors indicate *csf1* wild-type and knock-out fish, respectively. Each single dot represents one behavioral experiment, the center line indicates the median, box limits indicate the upper and lower quartiles, the whiskers indicate 1.5x interquartile range, and the points are outliers. The $P$-values (*P < 0.05; NS, not significant) are calculated using the Wald test in GLMMs. Source data are provided as a Source Data file.

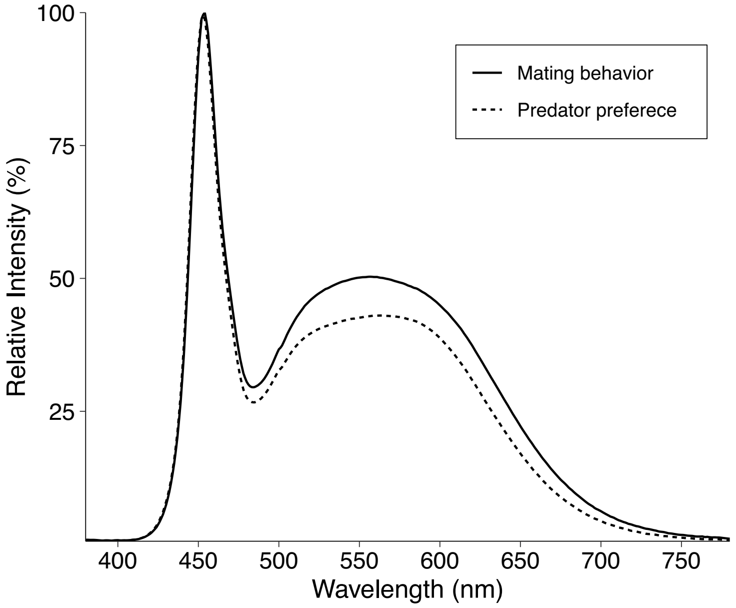


**Supplementary Figure 17 | Spectral curves of light sources used in the behavioral assay.** The relative intensity at each wavelength is shown for white LED lights used for the mating behavior assay (solid line) and the predator preference assay (dashed lines). These light sources cover the wavelength from 450 to 750 nm.

**Supplementary Table 1** List of species used for whole-genome sequencing

| Species | Collection site | ID | Sex | Accession No. | # of reads | # of bases |
|---|---|---|---|---|---|---|
| *A. oophorus* | Lake Poso, Central Sulawesi | | Female | DRR240750 | 120,287,878 | 18,163,469,578 |
| *A. poptae* | Lake Poso, Central Sulawesi | | Female | DRR240751 | 115,016,438 | 17,367,482,138 |
| *O. asinua* | Asinua River, Southeast Sulawesi | | Female | DRR240752 | 184,587,288 | 27,872,680,488 |
| *O. celebensis* | Asanae River, South Sulawesi | | Female | DRR240753 | 163,113,202 | 24,630,093,502 |
| *O. celebensis* | Malino River, South Sulawesi | | Female | DRR240754 | 227,182,766 | 34,304,597,666 |
| *O. dopingdopingensis* | Doping-doping River, South Sulawesi | | Female | DRR240755 | 180,255,330 | 27,218,554,830 |
| *O. eversi* | Tilanga Fountain, Central Sulawesi | | Female | DRR240756 | 107,417,198 | 16,219,996,898 |
| *O. hadiatyae* | Lake Masapi, South Sulawesi | | Female | DRR240757 | 126,025,518 | 19,029,853,218 |
| *O. marmoratus* | Lake Towuti, South Sulawesi | A | Female | DRR240758 | 146,684,504 | 22,149,360,104 |
| *O. marmoratus* | Lake Towuti, South Sulawesi | B | Female | DRR240759 | 107,134,412 | 16,177,296,212 |
| *O. marmoratus* | Lake Lantoa, South Sulawesi | | Female | DRR240760 | 148,261,848 | 22,387,539,048 |
| *O. marmoratus* | Lake Mahalona, South Sulawesi | | Female | DRR240761 | 165,975,626 | 25,062,319,526 |
| *O. matanensis* | Lake Matano, South Sulawesi | | Female | DRR240762 | 110,531,752 | 16,690,294,552 |
| *O. nebulosus* | Lake Poso, Central Sulawesi | | Female | DRR240763 | 219,603,348 | 33,160,105,548 |
| *O. nigrimas* | Lake Poso, Central Sulawesi | | Female | DRR240764 | 114,494,572 | 17,288,680,372 |
| *O. orthognathus* | Lake Poso, Central Sulawesi | | Female | DRR240765 | 224,211,496 | 33,855,935,896 |
| *O. profundicola* | Lake Towuti, South Sulawesi | | Female | DRR240766 | 142,532,956 | 21,522,476,356 |
| *O. sarasinorum* | Lake Lindu, Central Sulawesi | | Female | DRR240767 | 121,214,518 | 18,303,392,218 |
| *O. soerotoi* | Lake Tiu, Central Sulawesi | | Female | DRR240768 | 140,353,036 | 21,193,308,436 |
| *O. wolasi* | Anduna River, Southeast Sulawesi | | Female | DRR240769 | 184,482,342 | 27,856,833,642 |
| *O. wolasi* | Moramo Waterfall, Southeast Sulawesi | | Female | DRR240770 | 94,178,320 | 14,220,926,320 |
| *O. wolasi* | Moramo River, Southeast Sulawesi | | Female | DRR240771 | 71,995,676 | 10,871,347,076 |
| *O. woworae* | Fotuno Fountain, Muna Island | | Female | DRR240772 | 194,524,210 | 19,646,945,210 |
| *O. woworae* | Balano Fountain, Muna Island | | Female | DRR240773 | 176,177,564 | 26,503,740,060 |
| *O. woworae* | Laweau River, Muna Island | | Female | DRR240774 | 75,705,520 | 11,431,533,520 |
| *O. woworae* | Motobano Fountain, Muna Island | | Female | DRR240775 | 64,361,158 | 9,718,534,858 |

**Supplementary Table 2** Summary of quantitative trait locus (QTL) analysis

| Trait | LG | Position (cM) | 95% BI (cM) | Nearest Maker | LOD | Threshold* | PVE (%) | P-value | Additive Effect (a)† | Dominace Effect (d) | Dominance (\|d/a\|) |
|---|---|---|---|---|---|---|---|---|---|---|---|
| Sex | 24 | 53.03 | 50.24–55.83 | chr24:15,026,763 | 18.77 | 3.77 | 40.97 | < 0.001 | 3.1433 | 0.2647 | 0.084210861 |
| SL | 12_20_13 | 117.00 | 68.01–131.58 | chr12_20_13:66,117,828 | 5.089 | 3.79 | 13.32 | 0.003 | -1.45284 | 0.04081 | 0.02808981 |
| Pectoral fin (binary)§ | 7 | 41.42 | 34.19–65.23 | chr7:14,668,680 | 7.834 | 3.88 | 16.68 | < 0.001 | 6.8700 | 5.8050 | 0.844978166 |
| Pectoral fin (S1.Red)§ | 7 | 45.00 | 32.14–54.46 | chr7:16,239,469 | 5.228 | 3.77 | 11.45 | 0.002 | 0.0186 | 0.0343 | 1.843224663 |
| Pectoral fin (S1.Yellow)§ | 7 | 45.00 | 33.71–54.46 | chr7:16,239,469 | 5.976 | 3.76 | 13.18 | < 0.001 | 0.0108 | 0.0228 | 2.118886311 |
| Pectoral fin (S1.Blue)§ | 7 | 34.19 | 27.21–55.26 | chr7.12,610,865 | 4.216 | 3.83 | 9.289 | 0.023 | -0.0152 | -0.0348 | 2.295949334 |
| Lateral body (S1.Red)§ | 24 | 23.00 | 10.44–42.98 | chr24:6,312,988 | 3.869 | 3.83 | 10.2938 | 0.044 | -0.0133 | -0.0237 | 1.788802535 |
| Stripe (binary)§ | 12_20_13 | 77.20 | 74.22–92.48 | chr12_20_13:31,532,462 | 5.592 | 3.9 | 12.047 | 0.001 | -1.3366 | 1.2167 | 0.910294778 |
| Stripe (binary)§ | 15 | 53.00 | 51.48–59.49 | chr15:18,986,124 | 4.773 | 3.9 | 9.448 | 0.007 | -1.2227 | 0.8576 | 0.701398544 |

*LOD significance thresholds were determined by 1,000 permitaions ($\alpha = 0.05$).

†An additive effect is shown positive when an allele derived from the O. woworae parent yeilds a greater phenotypic value.

§Sex was included as an additive covariate.

LG, Linkage group; cM, centi Morgan; BI, Bayesian credible interval; LOD, logarithm of the odds; PVE, percentage of the phenotypic variance explained by a QTL.

**Supplementary Table 3** RNA-seq results of five candidate genes for red coloration of the pectoral fins.

| | | | | | *O. woworae* Males vs Females | | | | *O. woworae* Males vs *O. asinua* Males | | | |
|---|---|---|---|---|---|---|---|---|---|---|---|---|
| **Gene ID** | **Ensembl gene ID of BLAST top-hit** | **Gene name** | **Gene description** | **Genomic position** | **logFC*** | **logCPM** | **P-value** | **FDR** | **logFC†** | **logCPM** | **P-value** | **FDR** |
| G24311 | ENSOMEG00000010916 | *slmapb* | sarcolemma associated protein b | chr7: 14,747,711–14,760,520 | 2.04955 | 4.94635 | 6.22E-12 | 3.23E-09 | 1.91374 | 4.79235 | 2.64E-09 | 7.97E-07 |
| G24299 | ENSOMEG00000010499 | *csf1* | colony stimulating factor 1 | chr7: 14,881,558–14,867,637 | 1.88924 | 5.07566 | 1.83E-09 | 4.82E-07 | 2.78648 | 4.74989 | 2.56E-12 | 1.79E-09 |
| G24293 | ENSOMEG00000010462 | *ren* | renin | chr7: 14,896,848–14,892,323 | 3.32169 | 1.89912 | 1.84E-06 | 2.26E-04 | 4.34706 | 1.7944 | 1.04E-06 | 1.60E-04 |
| G24364 | ENSOMEG00000010364 | *magi1a* | membrane associated guanylate kinase | chr7: 14,916,412–15,001,460 | 1.55716 | 4.80449 | 1.09E-05 | 9.30E-04 | 1.74981 | 4.58028 | 6.45E-07 | 1.05E-04 |
| G24276 | ENSOMEG00000011545 | *pcbp4* | poly(rC) binding protein 4 | chr7: 14,520,992–14,554,119 | 1.51893 | 3.24485 | 1.30E-04 | 5.92E-03 | 1.90515 | 3.00857 | 3.24E-05 | 2.44E-03 |

*The value is positive when the expression levels are higher in males than in females.

†The value is positive when expression levels are higher in O. woworae than in O. asinua.

logFC, logarithm of fold change between groups; logCPM, logarithm of count per million reads; FDR, false discovery rate.

**Supplementary Table 4** Statistical results of Tukey's post hoc test for *csf1* expression levels in the $F_2$ family (related to Figure 3h)

| | | | 95% CI for difference | | |
|---|---|---|---|---|---|
| **Group 1** | **Group 2** | **Mean difference** | **Lower bound** | **Upper bound** | **Adjusted *P*** |
| C/W Male | C/C Male | 0.8034 | -0.0710 | 1.6777 | 0.0878 |
| W/W Male | C/C Male | 1.3851 | 0.5107 | 2.2594 | 0.0003 |
| C/C Female | C/C Male | 0.2784 | -0.5960 | 1.1527 | 0.9307 |
| C/W Female | C/C Male | 0.0048 | -0.8696 | 0.8791 | 1.0000 |
| W/W Female | C/C Male | 0.4429 | -0.4315 | 1.3172 | 0.6586 |
| W/W Male | C/W Male | 0.5817 | -0.2927 | 1.4561 | 0.3672 |
| C/C Female | C/W Male | -0.5250 | -1.3993 | 0.3494 | 0.4814 |
| C/W Female | C/W Male | -0.7986 | -1.6729 | 0.0758 | 0.0910 |
| W/W Female | C/W Male | -0.3605 | -1.2348 | 0.5139 | 0.8193 |
| C/C Female | W/W Male | -1.1067 | -1.9810 | -0.2323 | 0.0061 |
| C/W Female | W/W Male | -1.3803 | -2.2547 | -0.5059 | 0.0004 |
| W/W Female | W/W Male | -0.9422 | -1.8165 | -0.0678 | 0.0281 |
| C/W Female | C/C Female | -0.2736 | -1.1480 | 0.6008 | 0.9353 |
| W/W Female | C/C Female | 0.1645 | -0.7099 | 1.0389 | 0.9930 |
| W/W Female | C/W Female | 0.4381 | -0.4363 | 1.3125 | 0.6687 |

CI, credible interval

**Supplementary Table 5** Oligonucleotide sequences used in this study.

| Name | Sequence (5'-3') | Usage |
|---|---|---|
| csf1-qPCR-FW | AGCCAGAGAGCTTTGAGGTTCTT | qPCR |
| csf1-qPCR-RV | TCTGAAGCTTTCTGCAGTGCTT | qPCR, allele-specific expression |
| rpl13a-qPCR-FW | ACACGAGGTCGGCTGGAA | qPCR |
| rpl13a-qPCR-RV | TTGGCCTTCTCTTTTCTCTTCTCT | qPCR |
| csf1-exon2-FW | CCTGAGCTTCCCGCTGGTA | allele-specific expression |
| sgRNA-csf1_1 | TAATACGACTCACTATAGGGACACAAGGATGGTCATCGTTTTAGAGCTAGAA | Synthesis of sgRNA for csf1 #1 |
| sgRNA-csf1_2 | TAATACGACTCACTATAGGTGACTCAGGTTTGACACAGTTTTAGAGCTAGAA | Synthesis of sgRNA for csf1 #2 |
| sgRNA-RV | AAAAGCACCGACTCGGTGCC | sgRNA synthesis |
| csf1-exon1-HMA-FW | TTCTGTGGAGTGGAAGTGGA | HMA |
| csf1-exon1-HMA-RV | TTCAAAAGACGCTCACCTACC | HMA |

qPCR, quantitative PCR; sgRNA, single guide RNA; HMA, heteroduplex mobility assay
