# Supplementary material for: Genome editing reveals fitness effects of a gene for sexual dichromatism in Sulawesian fishes
Source: Nat Commun. 2021 Mar 1;12:1350. doi: 10.1038/s41467-021-21697-0 (PMC7921647; doi:10.1038/s41467-021-21697-0)
Supplement: Supplementary file 3 — Reporting Summary [file 41467_2021_21697_MOESM3_ESM.pdf]

nature research

Corresponding author(s): Jun Kitano

Last updated by author(s): Jan 26, 2021

Reporting Summary

Nature Research wishes to improve the reproducibility of the work that we publish. This form provides structure for consistency and transparency in reporting. For further information on Nature Research policies, see our Editorial Policies and the Editorial Policy Checklist.

Statistics

For all statistical analyses, confirm that the following items are present in the figure legend, table legend, main text, or Methods section.

n/a	Confirmed	
☐	☒	The exact sample size (*n*) for each experimental group/condition, given as a discrete number and unit of measurement
☐	☒	A statement on whether measurements were taken from distinct samples or whether the same sample was measured repeatedly
☐	☒	The statistical test(s) used AND whether they are one- or two-sided<br>*Only common tests should be described solely by name; describe more complex techniques in the Methods section.*
☐	☒	A description of all covariates tested
☐	☒	A description of any assumptions or corrections, such as tests of normality and adjustment for multiple comparisons
☐	☒	A full description of the statistical parameters including central tendency (e.g. means) or other basic estimates (e.g. regression coefficient) AND variation (e.g. standard deviation) or associated estimates of uncertainty (e.g. confidence intervals)
☐	☒	For null hypothesis testing, the test statistic (e.g. *F*, *t*, *r*) with confidence intervals, effect sizes, degrees of freedom and *P* value noted<br>*Give P values as exact values whenever suitable.*
☒	☐	For Bayesian analysis, information on the choice of priors and Markov chain Monte Carlo settings
☐	☒	For hierarchical and complex designs, identification of the appropriate level for tests and full reporting of outcomes
☐	☒	Estimates of effect sizes (e.g. Cohen's *d*, Pearson's *r*), indicating how they were calculated

Our web collection on statistics for biologists contains articles on many of the points above.

Software and code

Policy information about availability of computer code

Data collection	For the behavioral analysis, the position of the fish was extracted using DeepLabCut v2.1.6.4 installed on Google Colaboratory.
Data analysis	For sequence assembly, we used Falcon v0.7, Falcon Unzip v0.4.0., BWA-MEM v0.7.7, samtools v0.1.9, Bionano Solve v3.1, BUSCO v3.1.0, Trim Galore 0.4.3, Cutadapt 1.12, STAMPY v1.0.32, samtools v1.7, bcftools v1.7, R package onemap, R package qtl, ALLMAPS, RepeatModeler v1.0.11, RepeatMasker v4.0.7, and RMBlast v2.6.0. For gene annotation, we used Trim Galore 0.4.3, Cutadapt 1.12, HISAT2 v2.1.0, samtools, StringTie v1.3.5, TACO v0.7.3, TransDecoder v5.5.0, BUSCO v3.1.0, and OrthoFinder v2.2.6. For phylogenomic analysis, we used Trim Galore 0.4.4_dev , Cutadapt 1.17, BWA-MEM v0.7.17, Picard Tools, OrthoFinder v2.3.11, VCFtools v0.1.16, a custom script (https://github.com/satoshi-ansai/genome_utils/blob/master/vcf-tab_to_fasta.py) written with Python 3.7.6, MACSE v2.03, AMAS concat, IQ-TREE v1.6.12, FigTree v1.4.4, and Adobe Illustrator 2020. For divergence time estimation, we used SortaDate, RAxML v8.2.12, and MEGA X. For QTL analysis, the R package qtl was used. For RNA-seq of differentially expressed genes, we used Trim Galore 0.4.3, Cutadapt 1.12, STAR v2.6.1d, featureCounts v1.6.3, and edgeR v3.22.5. For phylogenetic analysis of csf1 gene, we used ORTHOSCOPE v1.0.2, MAFFT v7.453, PAL2NAL v14, trimAl v1.4.rev15, and iqtree v1.6.12. For allele-specific expression analysis, we used ApE v2.0.53, Adobe Illustrator CS6, Adobe Photoshop CS6, and R package exactRankTests. For statistical analysis of behavior, R packages trajr version 1.3.0, lme4 version 1.1-23, lmerTest version 3.1-2, and emmeans version 1.4.7 were used. The reflectance was analyzed with the R package pavo. For identification of androgen response elements, we used Canu v1.7, pbalign v0.3.1, arrow v2.2.2, BWA-MEM v0.7.17, Pilon v1.22, HOMER v4.11, BLASTN v2.6.0+, and Easyfig v2.2.5. For qPCR primer design, we used Primer Express 3.0. The brightness and contrast of microscopic images were adjusted with Fiji of ImageJ.

For manuscripts utilizing custom algorithms or software that are central to the research but not yet described in published literature, software must be made available to editors and reviewers. We strongly encourage code deposition in a community repository (e.g. GitHub). See the Nature Research guidelines for submitting code & software for further information.

Data

Policy information about availability of data

All manuscripts must include a data availability statement. This statement should provide the following information, where applicable:

- Accession codes, unique identifiers, or web links for publicly available datasets
- A list of figures that have associated raw data
- A description of any restrictions on data availability

All sequence reads are available from DDBJ: sequence reads used for de novo assembly of O. celebensis (DRA010635) and O. woworae (DRA011275), whole genome re-sequences (DRA010665); RNA-seq of multiple tissues for gene annotation (DRA010666); differential expression analysis by RNA-seq (DRA010667); ddRAD-seq reads for linkage mapping (DRA010679). Assembled reference sequence of O. celebensis (BNCR01000001-BNCR01000594) and O. woworae (BOLG01000001-BOLG01001666) are also available from DDBJ. Gene annotations for the reference assembly of O. celebensis are available from a GitHub repository (https://github.com/satoshi-ansai/OryCel_1.0/). The Ensembl protein databases (Release 94, http://oct2018.archive.ensembl.org/) were used for gene annotation: O. latipes (ASM223467v1), O. malastigma (Om_v0.7.RACA) and Danio rerio (GRCz11). The reference genome assemblies and their gene annotations in Ensembl Release 100 (http://apr2020.archive.ensembl.org/) were used for phylogenomic analysis: O. latipes (ASM223467v1), O. sakaizumii HNI (ASM223471v1), O. latipes HSOK (ASM223469v1), O. sinensis (ASM858656v1), O. malastigma (Om_v0.7.RACA), O. javanicus (OJAV_1.1), and Xiphophorus maculatus (X_maculatus-5.0-male). Source data are provided with this paper.

Field-specific reporting

Please select the one below that is the best fit for your research. If you are not sure, read the appropriate sections before making your selection.

☒ Life sciences ☐ Behavioural & social sciences ☐ Ecological, evolutionary & environmental sciences

For a reference copy of the document with all sections, see nature.com/documents/nr-reporting-summary-flat.pdf

Life sciences study design

All studies must disclose on these points even when the disclosure is negative.

Sample size	For genome sequencing, we used a single fish for each species to get the representative sequence. For QTL mapping, we used 164 F2 fish. It is generally recommended to use more than 200 individuals for QTL mapping (Li et al. 2010 Heredity), so we tried to make a family with as many progeny as possible and the maximum progeny that we could obtain was 164. For RNA-seq analysis, we used lab-raised fish with expected small variability and were interested in large differences in expression between species and sexes, so we used four individuals for each group as the recommended minimum number of replicate in previous publications (e.g. Lamarre et al. 2018 Front Plant Sci). For experiments to quantify the gene expression levels, sample sizes were determined based on the expected variability and the number of fish available according to other studies with similar methodology (e.g. Kusakabe et al. 2017 Mol Ecol; Ishikawa et al. 2016 Evo Eco Res). For behavioral experiments, we used as many fish as available.
Data exclusions	In the mate choice experiment, the pairs that did not spawn eggs during the 2h-test were excluded, because such female may not have been ready for spawning. In other experiments, no data were excluded.
Replication	Genome sequencing was conducted using one fish. QTL mapping was conducted using one family. For the gene expression analysis with RNA-seq and quantitative PCR, each experiment was conducted using multiple sample fish as biological replicates. All images and spectral values of the knock-in and knock-out fish were obtained from multiple fish ($n \geq 3$ in each group). Behavioral experiments were conducted with each fish as a replicate.
Randomization	In all behavioral experiments, each fish was randomly chosen from one of the families (i.e., family was randomized) and then allocated into the experimental group. In the behavioral experiments, each compartment was assigned randomly to the wild-type or the knockout fish.
Blinding	All genomic and behavioral experiments were conducted as blinded experiments.

Reporting for specific materials, systems and methods

We require information from authors about some types of materials, experimental systems and methods used in many studies. Here, indicate whether each material, system or method listed is relevant to your study. If you are not sure if a list item applies to your research, read the appropriate section before selecting a response.

Materials & experimental systems

n/a	Involved in the study
x	Antibodies
x	Eukaryotic cell lines
x	Palaeontology and archaeology
	x Animals and other organisms
x	Human research participants
x	Clinical data
x	Dual use research of concern

Methods

n/a	Involved in the study
x	ChIP-seq
x	Flow cytometry
x	MRI-based neuroimaging

Animals and other organisms

Policy information about studies involving animals; ARRIVE guidelines recommended for reporting animal research

Laboratory animals	The following fishes were used: Oryzias celebensis (Ujung pandang strain) (2 females; 1 male), O. celebensis (Malino strain) (1 female), O. woworae (Fotuno strain) (70 females; 69 males), O. asinua (Asinua strain) (12 females; 12 males), F2 hybrid between O. celebensis (Malino) and O. woworae (Fotuno) (73 females; 91 males), and Nomorhamphus cf. ebrardtii (Fotuno strain) (13 males). All used fish were at the adult stage (>3 months old after hatching). They were maintained in the aquarium at 26 C under the photoperiod of L:D = 14:10
Wild animals	No wild animals were used in this study. DNA samples collected in previous studies (Mokodongan and Yamahira 2015 Mol. Phylogenet. Evol. 93: 150-160) were used under the permit of the Ministry of Research, Technology, and Higher Education, Republic of Indonesia (RISTEKDIKTI) and the Faculty of Fisheries and Marine Science, Sam Ratulangi University (research permit numbers 394/SIP/FRP/SM/XI/2014, 397/SIP/FRP/SM/XI/2014, and 106/SIP/FRP/E5/Dit.KI/IV/2018).
Field-collected samples	No field collected samples were used in this study.
Ethics oversight	All animal experiments were conducted under approval by the Institutional Animal Care and Use Committee of the National Institute of Genetics (28-12, 27-5, and 26-15) and the National Institute for Basic Biology (19A055, 18A079, and 17A161).

Note that full information on the approval of the study protocol must also be provided in the manuscript.
